# Supplementary material for: SepF is the FtsZ anchor in archaea, with features of an ancestral cell division system
Source: Nat Commun. 2021 Jun 4;12:3214. doi: 10.1038/s41467-021-23099-8 (PMC8178401; doi:10.1038/s41467-021-23099-8)
Supplement: Supplementary file 1 — Supplementary Information [file 41467_2021_23099_MOESM1_ESM.pdf]

Supplementary information

SepF is the FtsZ anchor in archaea, with features of an ancestral cell division system

Pende N., Sogues A., *et al*. 2020

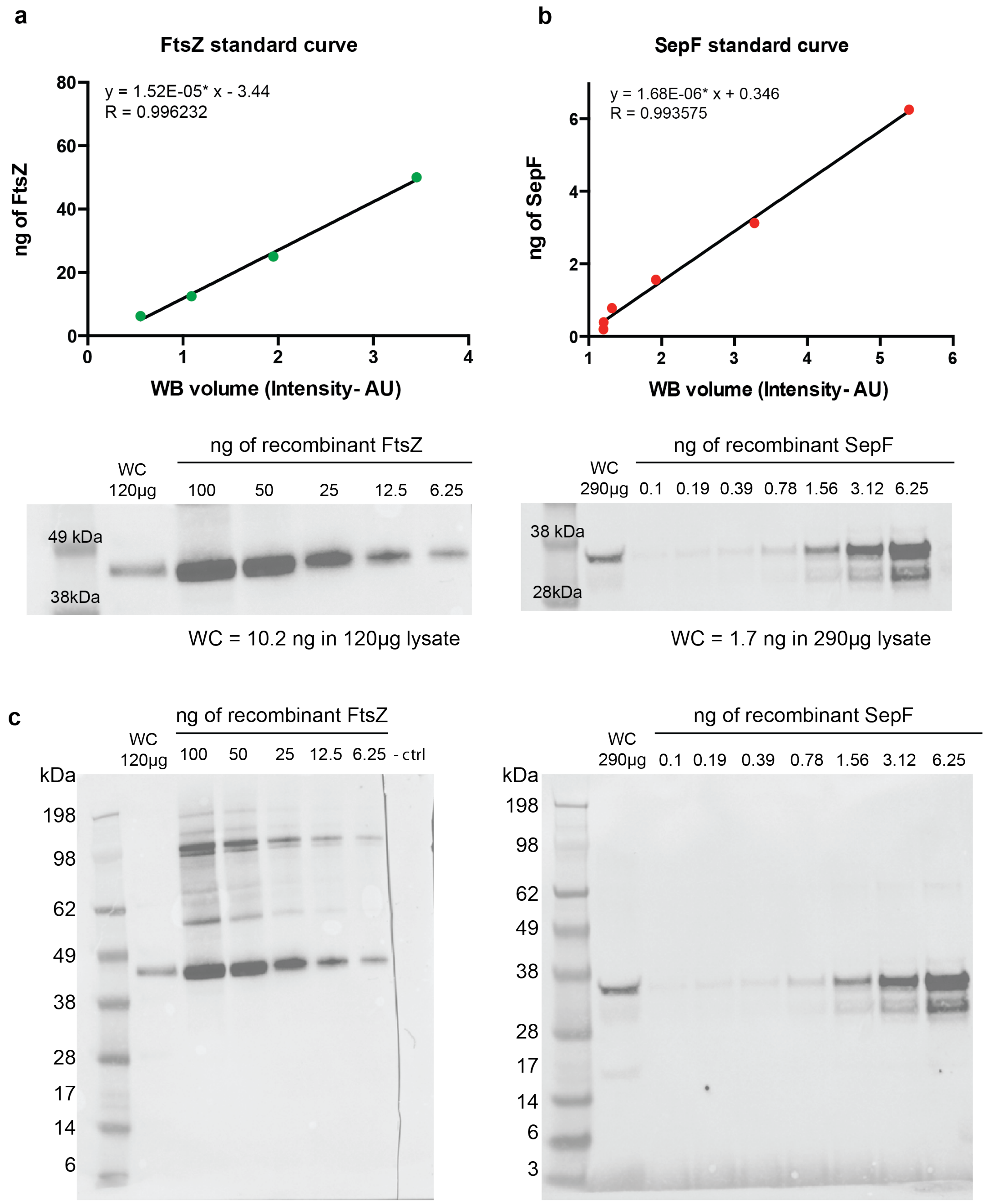

Supplementary Figure 1. Molar ratio of FtsZ/SepF in *M. smithii* and characterization of the specific anti-*Ms*FtsZ and anti-*Ms*SepF antibodies. a. Serial dilutions of recombinant FtsZ (100 ng to 6.25 ng). Band volumes were plotted against amount of FtsZ in order to calculate the linear regression. 120 µg of the whole cell extract in exponential phase were loaded and the total amount of FtsZ was calculated. **b.** Serial dilutions of recombinant SepF (6.25 ng to 0.1 ng). Band volumes were plotted against amount of SepF in order to calculate the linear regression. 290 µg of the whole cell extract in exponential phase were loaded and the total amount of SepF was calculated. **c.** Full uncropped Western Blots. Molecular weight markers (MW, in kDa) are shown on the side of the blot. The data shown here are representative for experiments performed at least twice.

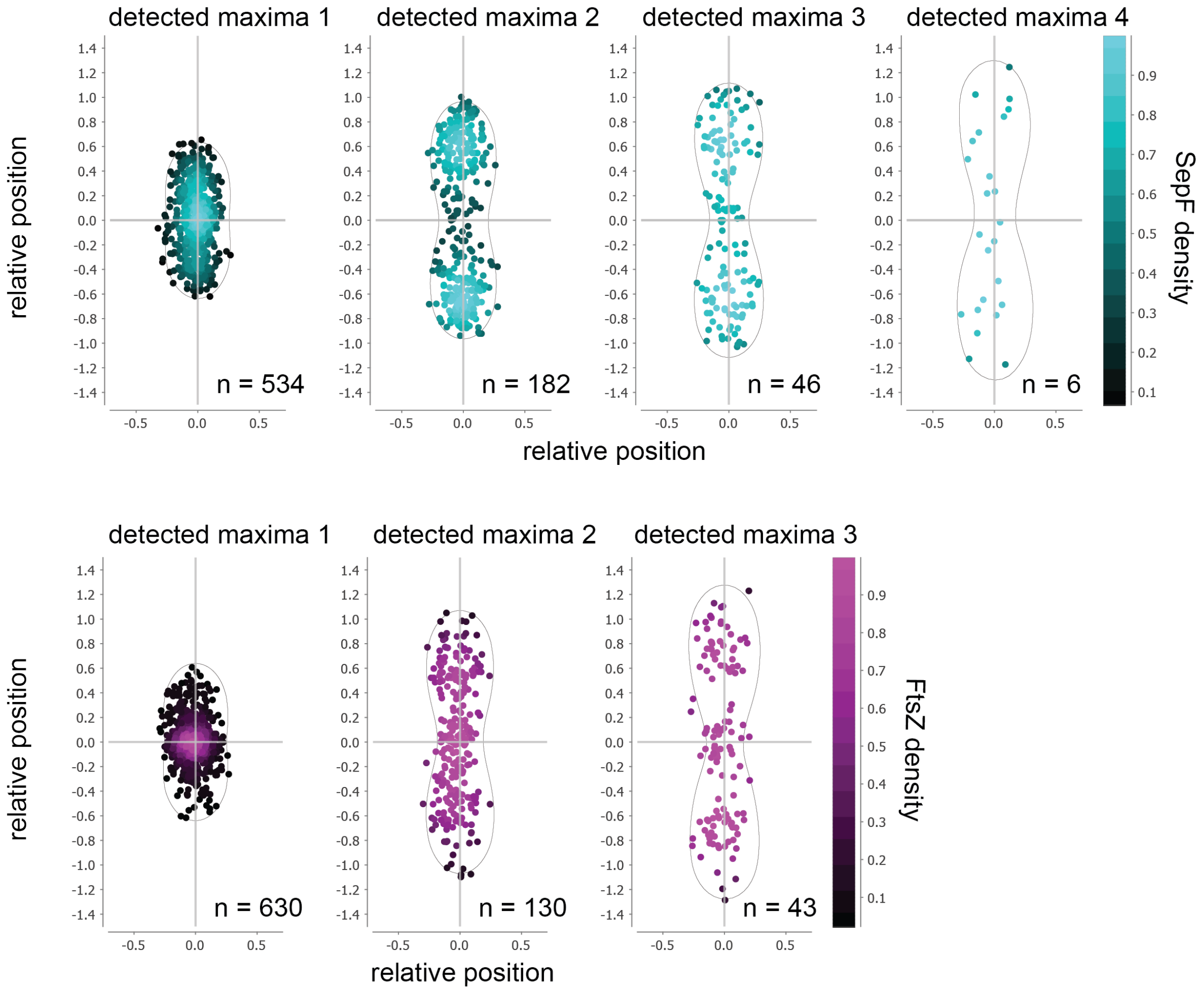

Supplementary Figure 2. Localization of SepF and FtsZ maxima within *M. smithii* cells. Relative position of detected fluorescent maxima within the cell grouped into four classes for SepF (maxima detected 1-4, upper panel) and three for FtsZ (maxima detected 1-3, lower panel). n, number of cells for each group. The data shown here are representative for experiments performed three times.

a

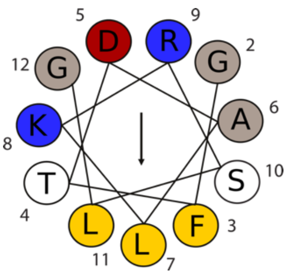

b

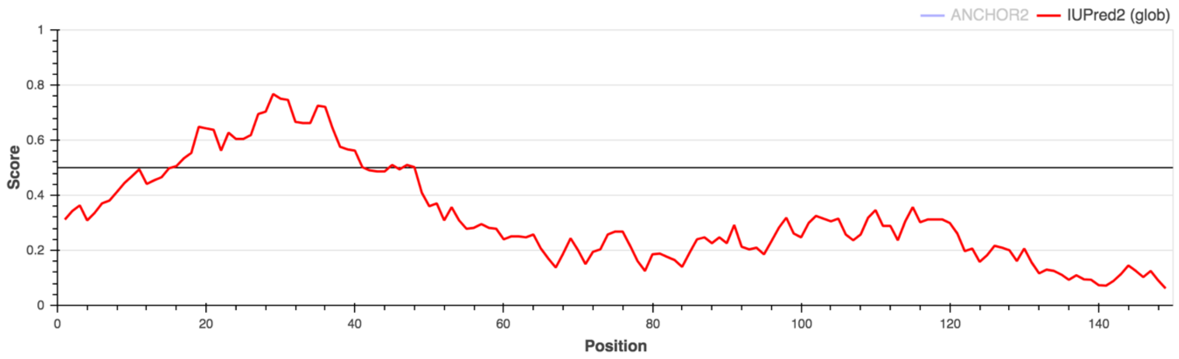

Supplementary Figure 3. **Functional and structural domains of SepF from *M. smithii.* a**. Predicted amphipathic helix for the M domain comprising amino acids from 2 to 12. Amino acid proprieties color code: gray (non-charged) yellow (apolar), red (negatively charged) and blue (positively charged). The prediction was made using HELIQUEST [1]. **b**. Intrinsically disordered region prediction of SepF was made using the IUPred2A software [2].

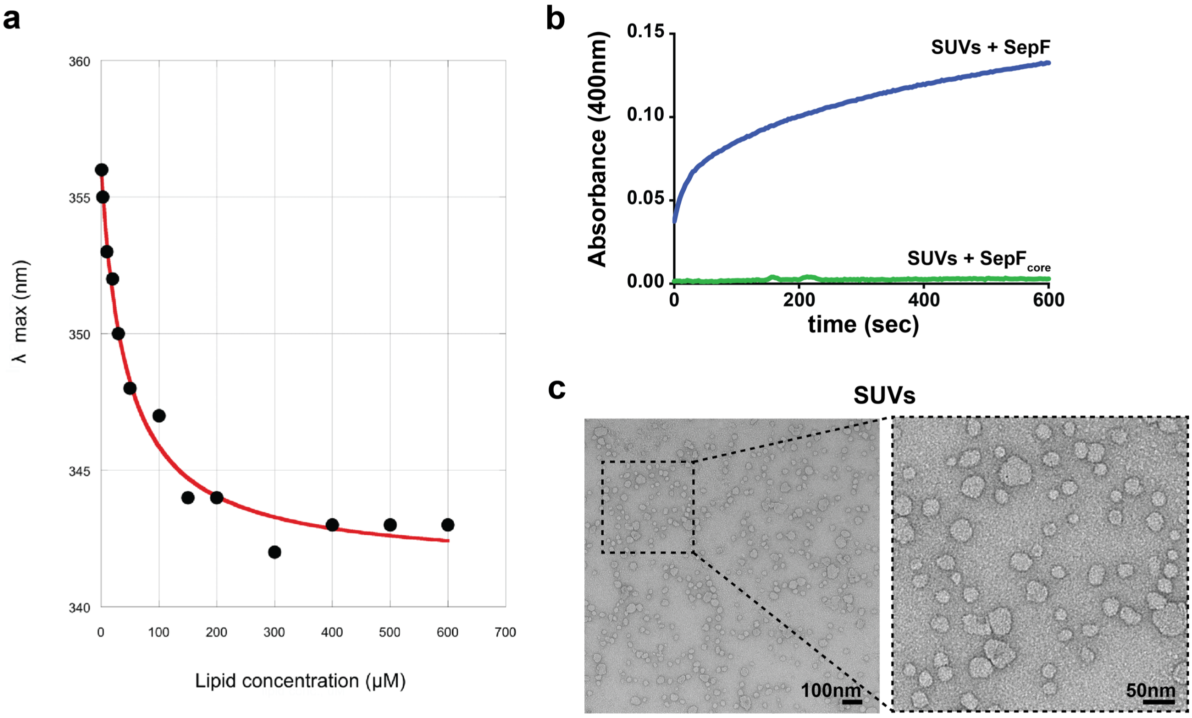

Supplementary Figure 4. SepF – Membrane interaction. a. Tryptophan fluorescence titration assay using a modified $SepF_M$ peptide (including a N-terminal tryptophan residue) as a function of lipid concentration (see Material and Methods for details). **b.** Polymerization assay for SepF (blue line) and $SepF_{core}$ (green line) in the presence of SUVs**. c.** Negative stain electron microscope image of SUVs (100 µmol L-1) alone. Panels show original image with an enlarged inlet that is marked by black dotted square next to it. Scale bar is 100 nm (original image) and 50 nm (inlet).

a

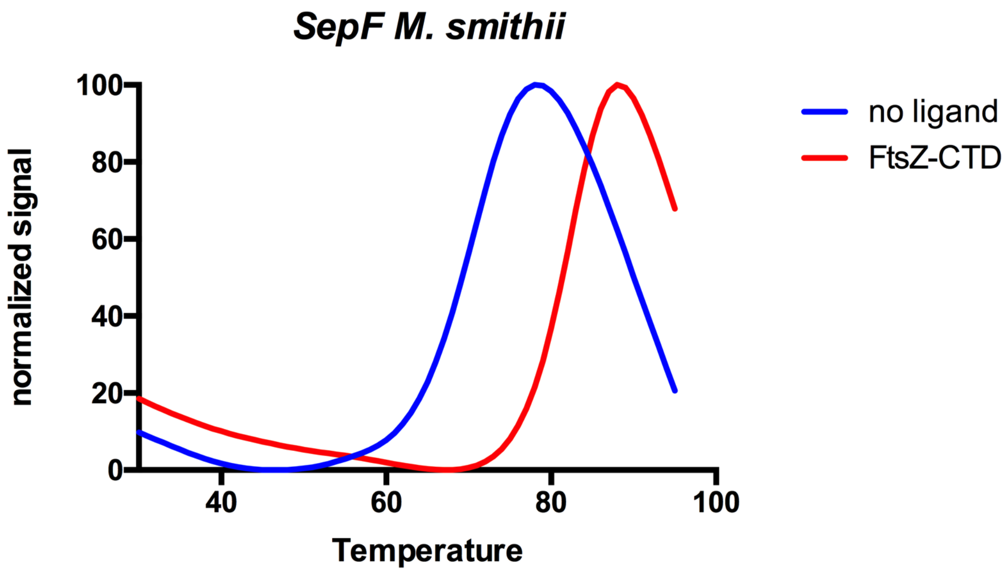

b

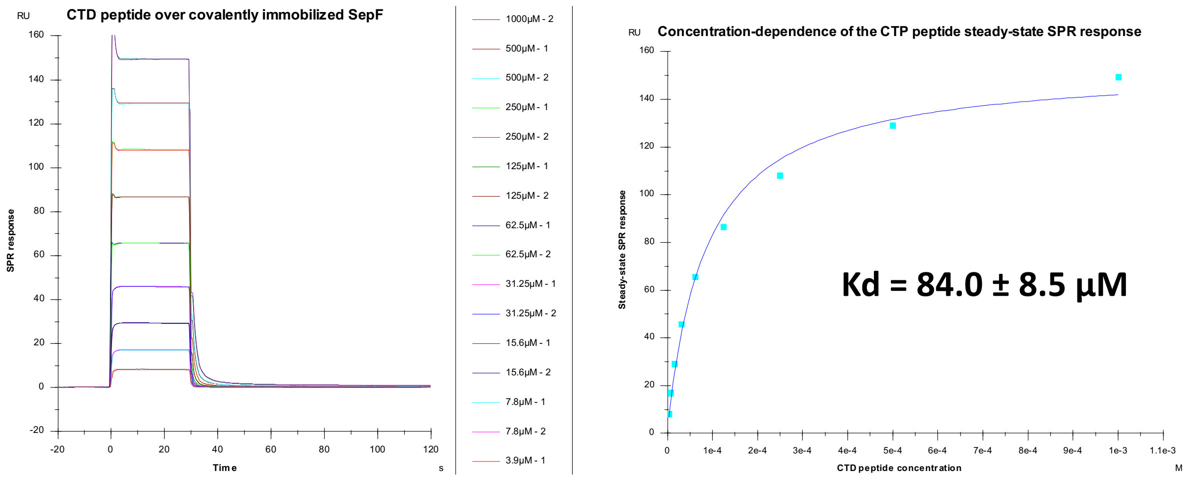

Supplementary Figure 5. *Ms*SepF-FtsZ$_{CTD}$ interactions. a. Thermal denaturation curves of *Ms*SepF$_{core}$ alone (blue) and upon addition of the FtsZ$_{CTD}$ peptide (red). The significant increase of protein thermostability (*Tm* values of 70.6 °C and 82.3 °C, respectively) indicates a strong interaction *in vitro*. Experiments were performed in triplicates. **b.** SPR analysis of the interaction between SepF$_{core}$ and FtsZ$_{CTD}$.

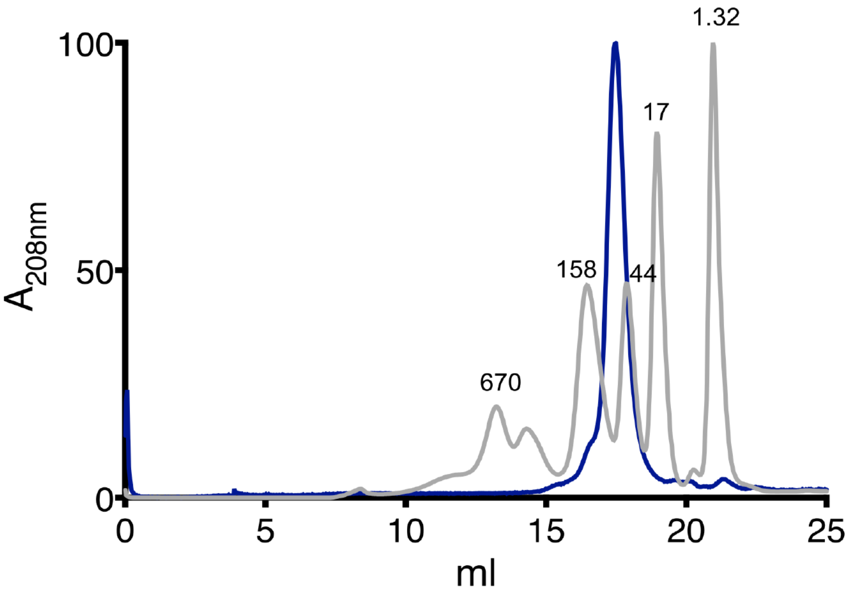

Supplementary Figure 6. Size exclusion chromatography of the full-length *Ms*SepF shows the dimeric state of the protein in solution. *Ms*SepF full-length (blue) on a Superdex S200 10/300 column. The molecular weight (MW) markers are shown in gray with corresponding MW indicated above. The data shown here are representative for experiments performed at least twice.

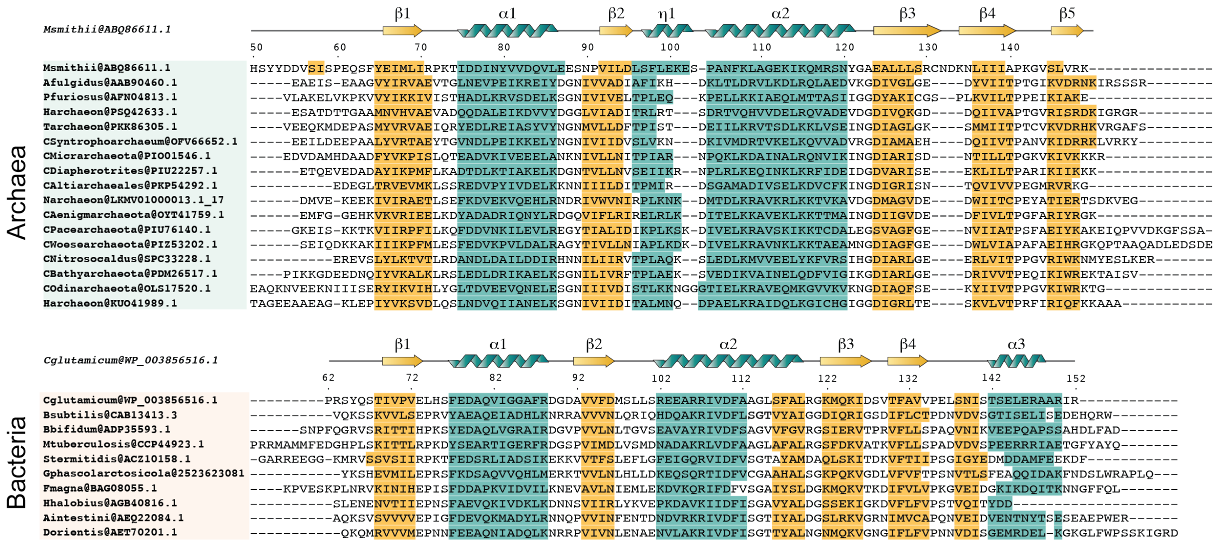

Supplementary Figure 7. **Secondary structure prediction of diverse representative linages in archaeal and bacterial SepF**. Turquoise indicates predicted α-helices whereas yellow indicates predicted β-strands. The experimental secondary structural elements as inferred from the crystal structures of *M. smithii* (this work) and *C. glutamicum* (PDB code 6SCP) are depicted above each sequence block. Prediction was performed using PSIPRED [3] and Ali2D [4]

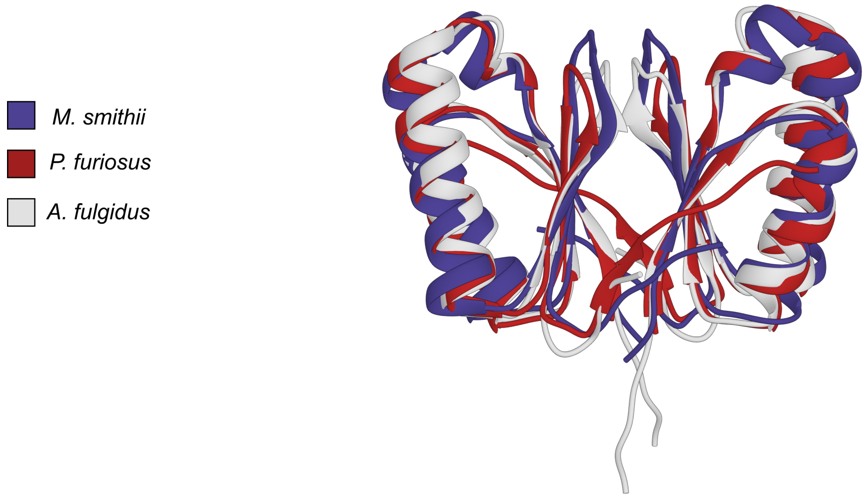

Supplementary Figure 8. Comparison of available archaeal SepF structures. Superposition of all the available archaeal SepF crystal structures (RMSDs of 1.5 - 1.9 Å for 75 – 77 aligned residues) show the same folding and dimer interface. The three structures contain the same secondary structural elements (α1 to α2, η1 and β1 to β5). *Archaeoglobus fulgidus* (PDB: 3ZIE), *Pyrococcus furiosus* (PDB: 3ZIG) [5].

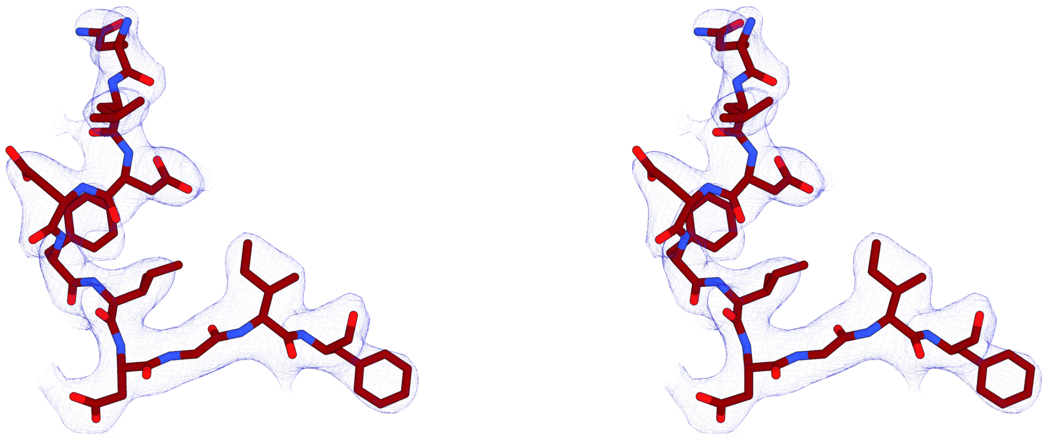

Supplementary Figure 9. Stereo representation of the electron density of the $FtsZ_{CTD}$ peptide bound to *Ms*SepF. Final (2Fo-Fc) electron density map countered at 1.3 σ.

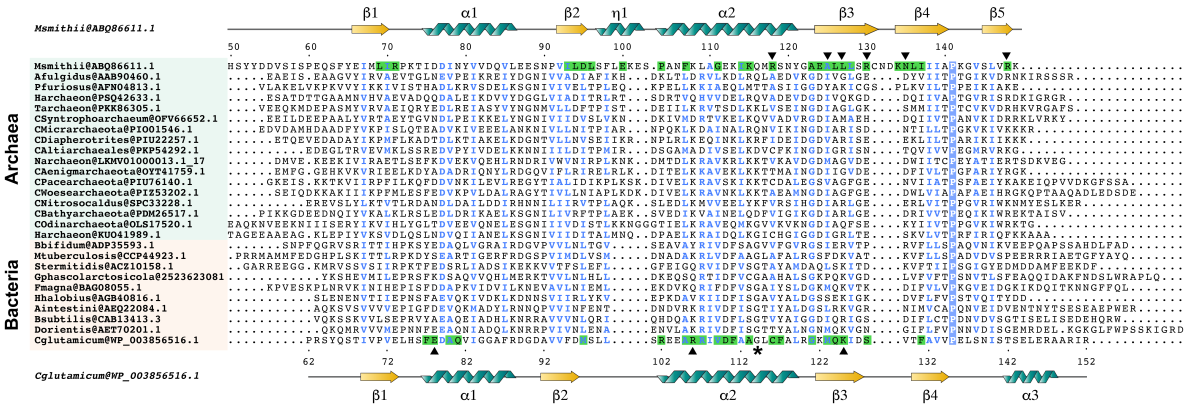

Supplementary Figure 10. Structure-based alignment of archaeal and bacterial SepF homologues. Sequences from diverse representative linages in Archaea and Bacteria were chosen and aligned based on structural features. SepF from *M. smithii* (this work) and from *C. glutamicum* (PDB: 6SCP) were chosen as structural models and their secondary structural elements are shown respectively above and below the alignment. Conserved positions are indicated in blue and an asterisk indicates the invariant Gly residue (Gly 114 in *Cg*SepF) that is crucial to form the dimer interface in bacterial homologs. For both crystal structures, $FtsZ_{CTD}$ contact residues (d < 5 Å) are highlighted in green, and those forming intermolecular hydrogen bonds are marked with a black triangle. Graphical representation was made using ENDscript server [6] and improved manually.

a

b

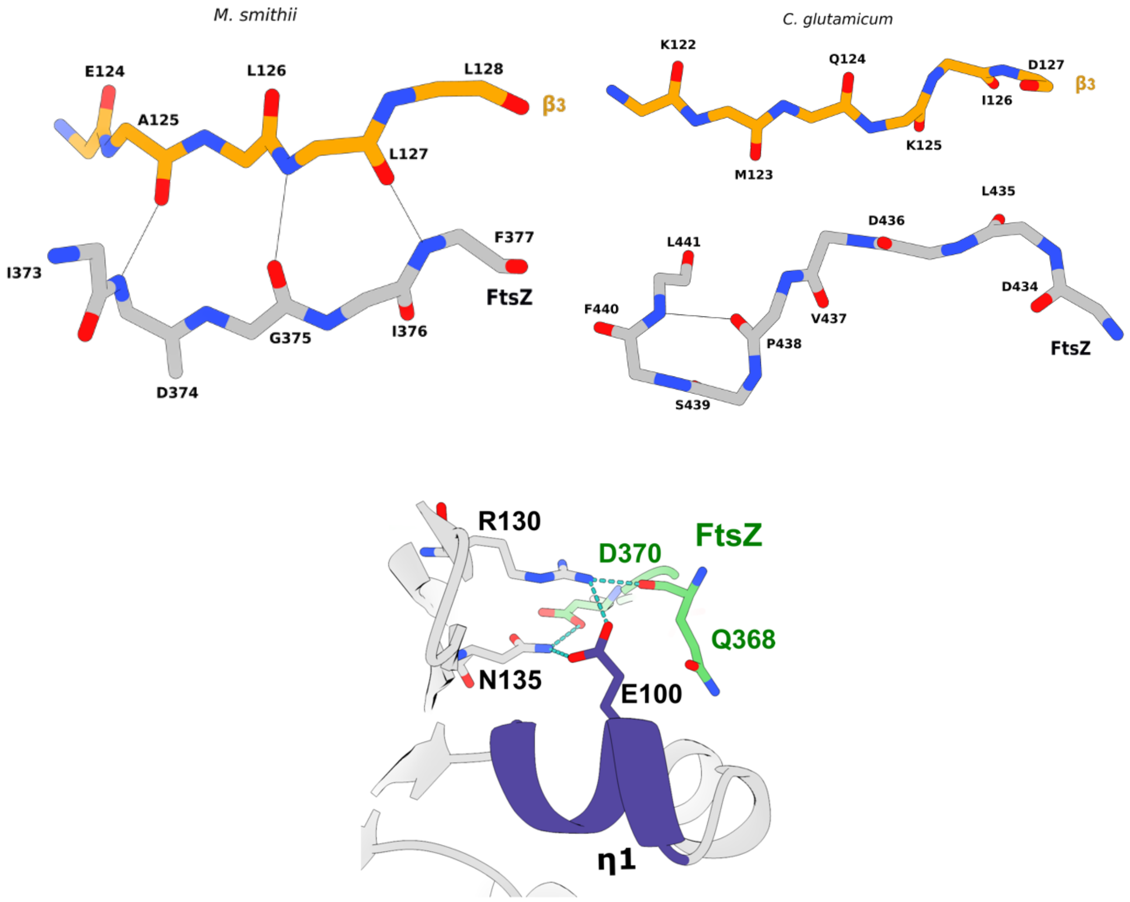

Supplementary Figure 11. **Structural details of FtsZ-SepF interactions**. **a**. Backbone interactions between the SepF strand β3 (yellow) and the $FtsZ_{CTD}$ main-chain atoms (grey) in *M. smithii* (left panel) and *C. glutamicum* (right panel). The FtsZ peptide adopts a different conformation and interacts differently with strand β3 in the two species. For clarity, H bonds are shown as black lines, and lateral side chains are omitted. **b**. Hydrogen bonding network involving the archaeal-specific η1 insertion (purple) in the *Ms*SepF-FtsZ complex.

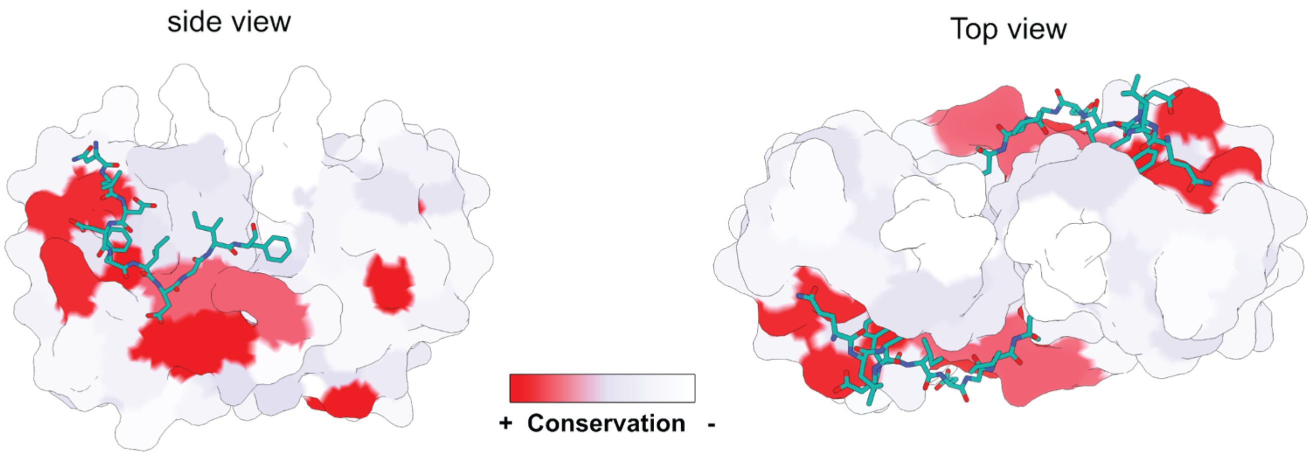

Supplementary Figure 12. Conservation of the FtsZ binding pocket in archaeal SepF. Conserved regions of archaeal SepF sequences were mapped on the $MsSepF_{core}$ structure using ConSurf-BD[7]. Red represents highly conserved residues, white indicates poorly conserved residues. Bound $FtsZ_{CTD}$ is shown in turquoise and stick representation.

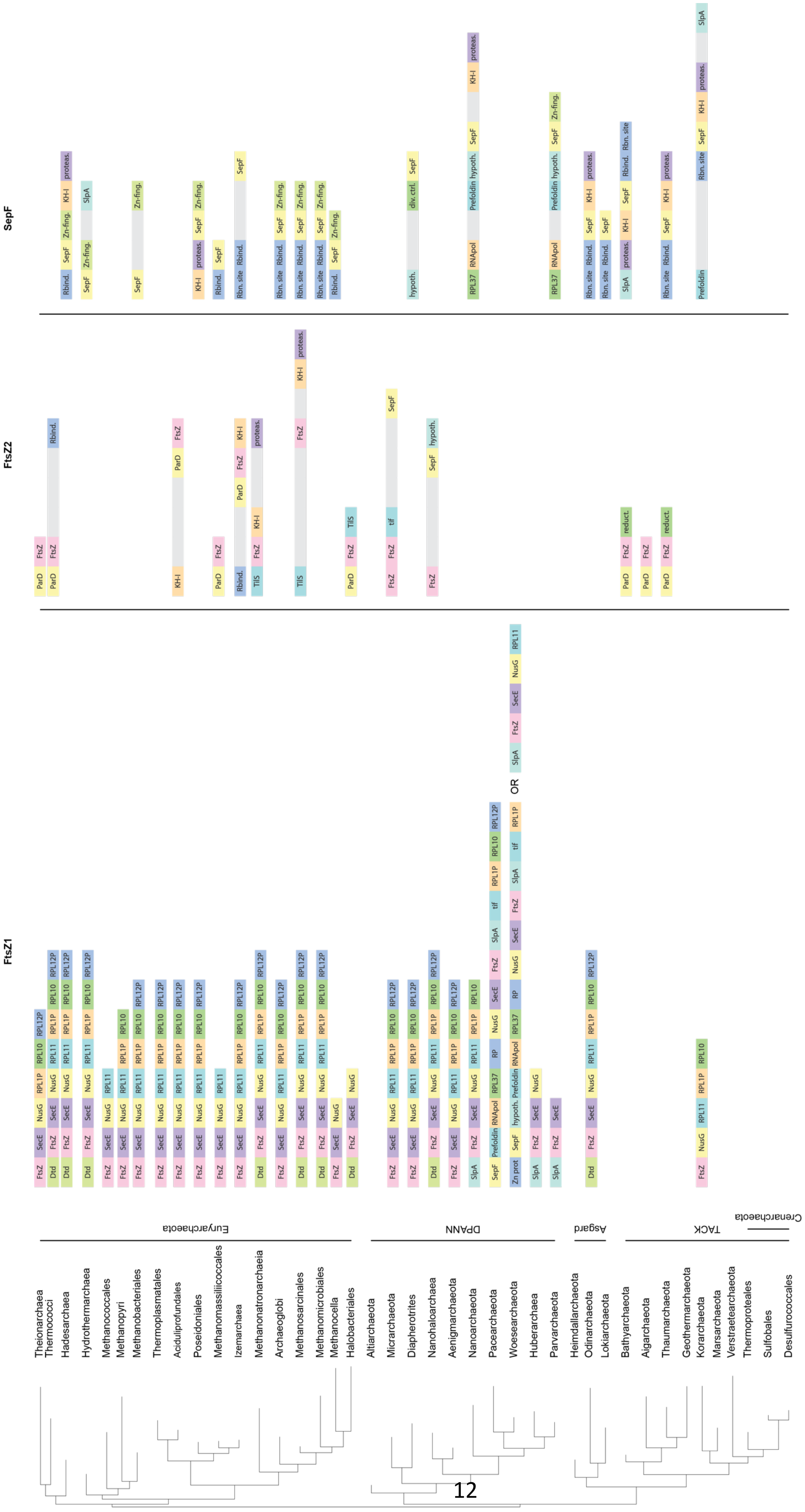
FtsZ1
FtsZ2
SepF
OR
Euryarchaeota
DPANN
Asgard
TACK
Crenarchaeota
Theionarchaea
Thermococci
Hadesarchaea
Hydrothermarchaea
Methanococcales
Methanopyri
Methanobacteriales
Thermoplasmatales
Aciduliprofundales
Poseidoniales
Methanomassiliicoccales
Izemarchaea
Methanonatronarchaeia
Archaeoglobi
Methanosarcinales
Methanomicrobiales
Methanocella
Halobacteriales
Altiarchaeota
Micrarchaeota
Diapherotrites
Nanohaloarchaeota
Aenigmarchaeota
Nanoarchaeota
Pacearchaeota
Woesearchaeota
Huberarchaea
Parvarchaeota
Heimdallarchaeota
Odinarchaeota
Lokiarchaeota
Bathyarchaeota
Aigarchaeota
Thaumarchaeota
Geothermarchaeota
Korarchaeota
Marsarchaeota
Verstraetearchaeota
Thermoproteales
Sulfobales
Desulfurococcales

Supplementary Figure 13. Genomic context of archaeal FtsZ1, FtsZ2 and SepF homologues mapped on a schematic reference phylogeny of the Archaea. The genomic context of the gene coding for FtsZ1 is well conserved in most archaeal lineages. Most of the genes around *ftsZ1* are involved in transcription, translation and regulation. In contrast, the genomic contexts of the genes coding for SepF and FtsZ2 are less conserved. In some members of the DPANN superphylum *sepF* can be found in the same conserved cluster as *ftsZ1* of *ftsZ2*, supporting the functional link between the two proteins.
div. ctrl.: ORC1-type DNA replication protein; dtd: D-aminoacyl-tRNA deacylase; FtsZ: FtsZ, hypoth.: Uncharacterized protein family (UPF0147); KH-I: K homology RNA-binding domain type I; proteas.: Proteasome subunit; Rbind.: RNA-binding protein; Rbn.: site RNA binding site; reduct.: Nitro FMN reductase; RNApol: DNA-directed RNA polymerase; RP: 50S ribosomal protein; RPL10: Ribosomal protein L10 family; RPL11: 50S ribosomal protein L11; RPL12P: 50S ribosomal protein L12P; RPL1P: 50S ribosomal protein L1P; RPL37: 50S ribosomal protein L37; tif: translation initiation factor IF-5A; Zn-fing: ZPR1 zinc-finger domain protein. Not conserved genes are represented in grey.

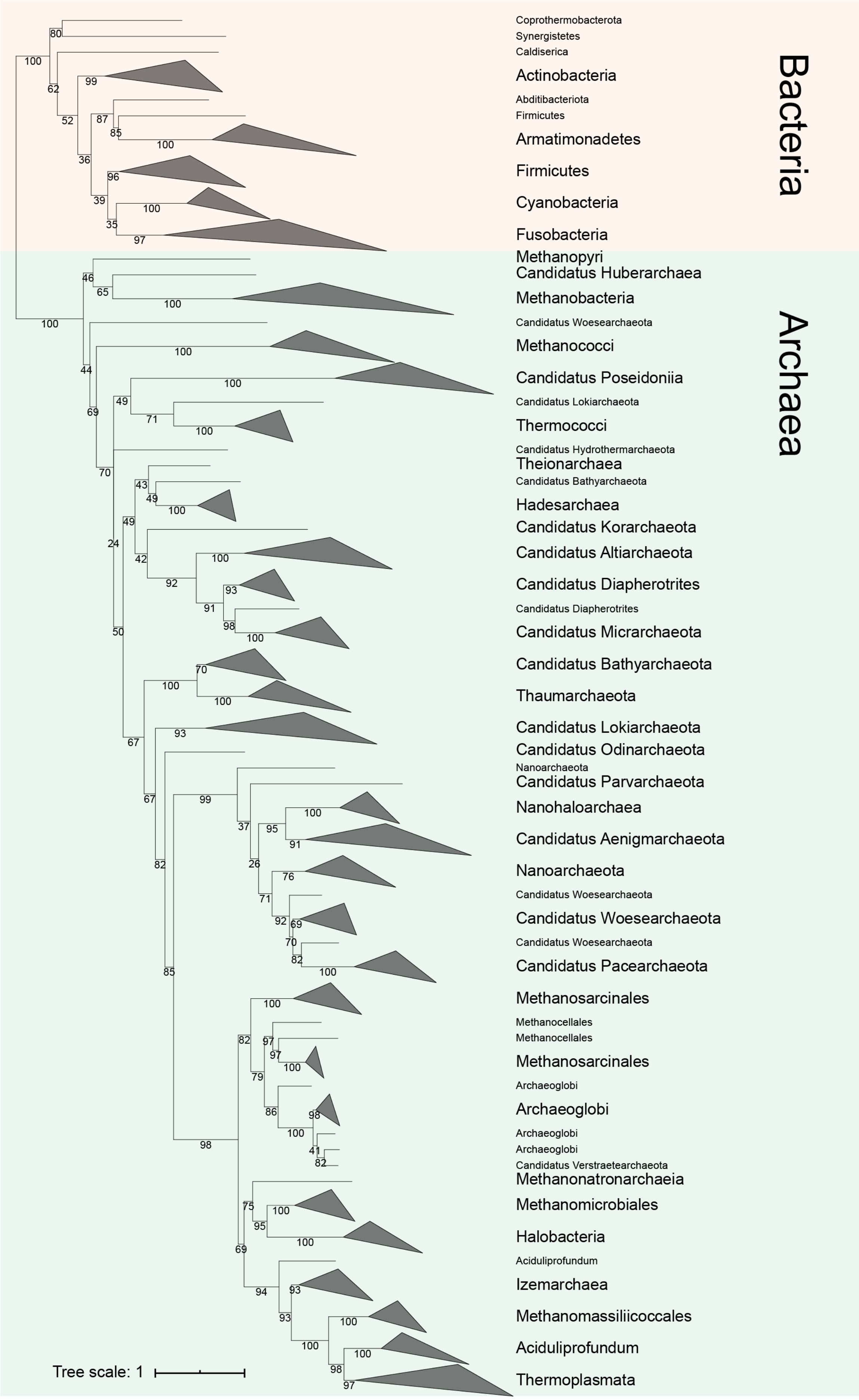
Coprothermobacterota
Synergistetes
Caldiserica
Actinobacteria
Abditibacteriota
Firmicutes
Armatimonadetes
Firmicutes
Cyanobacteria
Fusobacteria
Bacteria
Methanopyri
Candidatus Huberarchaea
Methanobacteria
Candidatus Woesearchaeota
Methanococci
Candidatus Poseidoniia
Candidatus Lokiarchaeota
Thermococci
Candidatus Hydrothermarchaeota
Theionarchaea
Candidatus Bathyarchaeota
Hadesarchaea
Candidatus Korarchaeota
Candidatus Altiarchaeota
Candidatus Diapherotrites
Candidatus Diapherotrites
Candidatus Micrarchaeota
Candidatus Bathyarchaeota
Thaumarchaeota
Candidatus Lokiarchaeota
Candidatus Odinarchaeota
Nanoarchaeota
Candidatus Parvarchaeota
Nanohaloarchaea
Candidatus Aenigmarchaeota
Nanoarchaeota
Candidatus Woesearchaeota
Candidatus Woesearchaeota
Candidatus Woesearchaeota
Candidatus Pacearchaeota
Methanosarcinales
Methanocellales
Methanocellales
Methanosarcinales
Archaeoglobi
Archaeoglobi
Archaeoglobi
Archaeoglobi
Candidatus Verstraetearchaeota
Methanonatronarchaeia
Methanomicrobiales
Halobacteria
Aciduliprofundum
Izemarchaea
Methanomassiliicoccales
Aciduliprofundum
Thermoplasmata
Archaea
Tree scale: 1

Supplementary Figure 14. Phylogeny of SepF homologues in Bacteria and Archaea. Maximum likelihood tree of SepF inferred with IQ-TREE v1.6.7.2 (ModelFinder best-fit model LG+F+R5)[8, 9] from an alignment of 147 sequences and 143 amino acid positions. Numbers at nodes represent ultrafast bootstrap supports [10]. The scale bar represents the average number of substitutions per site. There is a clear separation between Bacteria and Archaea and the tree roughly recapitulates known phylogenetic relationships. This result suggests that SepF was already present in the LUCA and followed a mainly vertical evolution in the two prokaryotic domains. While SepF was lost in many Bacteria, it was largely retained in Archaea.

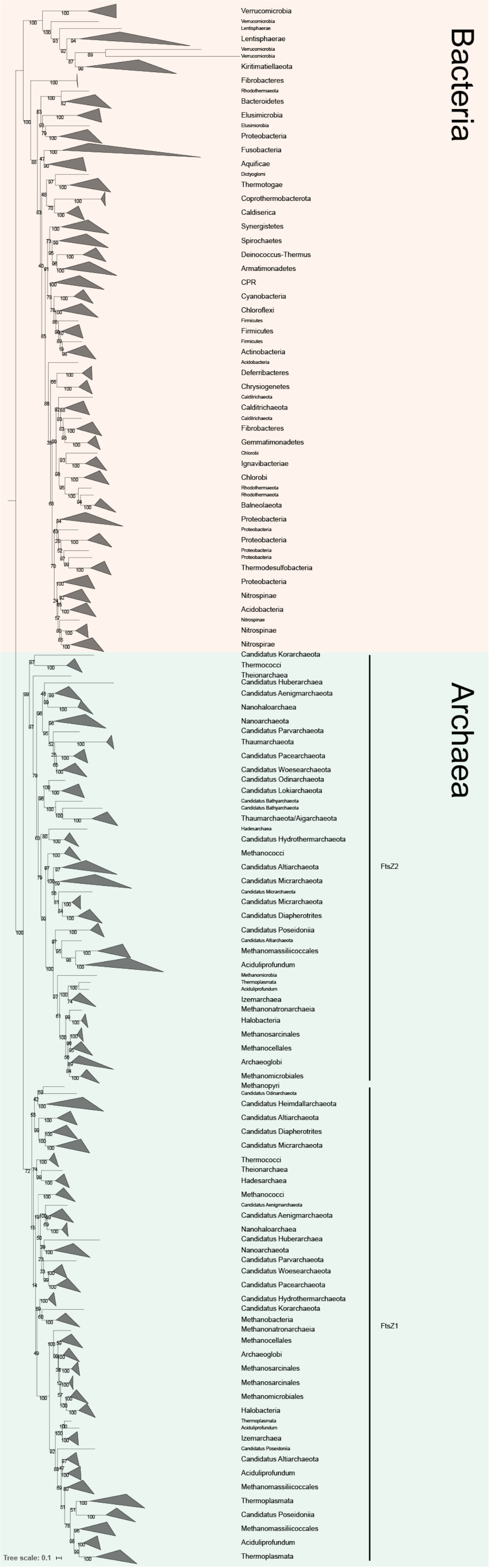
Bacteria
Verrucomicrobia
Verrucomicrobia
Lentisphaerae
Lentisphaerae
Verrucomicrobia
Verrucomicrobia
Kiritimatiellaeota
Fibrobacteres
Rhodothermaeota
Bacteroidetes
Elusimicrobia
Elusimicrobia
Proteobacteria
Fusobacteria
Aquificae
Dictyoglomi
Thermotogae
Coprothermobacterota
Caldiserica
Synergistetes
Spirochaetes
Deinococcus-Thermus
Armatimonadetes
CPR
Cyanobacteria
Chloroflexi
Firmicutes
Firmicutes
Firmicutes
Actinobacteria
Acidobacteria
Deferribacteres
Chrysiogenetes
Calditrichaeota
Calditrichaeota
Calditrichaeota
Fibrobacteres
Gemmatimonadetes
Chlorobi
Ignavibacteriae
Chlorobi
Rhodothermaeota
Rhodothermaeota
Balneolaeota
Proteobacteria
Proteobacteria
Proteobacteria
Proteobacteria
Proteobacteria
Thermodesulfobacteria
Proteobacteria
Nitrospinae
Acidobacteria
Nitrospinae
Nitrospinae
Nitrospirae
Archaea
Candidatus Korarchaeota
Thermococci
Theionarchaea
Candidatus Huberarchaea
Candidatus Aenigmarchaeota
Nanohaloarchaea
Nanoarchaeota
Candidatus Parvarchaeota
Thaumarchaeota
Candidatus Pacearchaeota
Candidatus Woesearchaeota
Candidatus Odinarchaeota
Candidatus Lokiarchaeota
Candidatus Bathyarchaeota
Candidatus Bathyarchaeota
Thaumarchaeota/Aigarchaeota
Hadesarchaea
Candidatus Hydrothermarchaeota
Methanococci
Candidatus Altiarchaeota
Candidatus Micrarchaeota
Candidatus Micrarchaeota
Candidatus Micrarchaeota
Candidatus Diapherotrites
Candidatus Poseidoniia
Candidatus Altiarchaeota
Methanomassiliicoccales
Aciduliprofundum
Methanomicrobia
Thermoplasmata
Aciduliprofundum
Izemarchaea
Methanonatronarchaeia
Halobacteria
Methanosarcinales
Methanocellales
Archaeoglobi
Methanomicrobiales
Methanopyri
Candidatus Odinarchaeota
Candidatus Heimdallarchaeota
Candidatus Altiarchaeota
Candidatus Diapherotrites
Candidatus Micrarchaeota
Thermococci
Theionarchaea
Hadesarchaea
Methanococci
Candidatus Aenigmarchaeota
Candidatus Aenigmarchaeota
Nanohaloarchaea
Candidatus Huberarchaea
Nanoarchaeota
Candidatus Parvarchaeota
Candidatus Woesearchaeota
Candidatus Pacearchaeota
Candidatus Hydrothermarchaeota
Candidatus Korarchaeota
Methanobacteria
Methanonatronarchaeia
Methanocellales
Archaeoglobi
Methanosarcinales
Methanosarcinales
Methanomicrobiales
Halobacteria
Thermoplasmata
Aciduliprofundum
Izemarchaea
Candidatus Poseidoniia
Candidatus Altiarchaeota
Aciduliprofundum
Methanomassiliicoccales
Thermoplasmata
Candidatus Poseidoniia
Methanomassiliicoccales
Aciduliprofundum
Thermoplasmata
FtsZ2
FtsZ1
Tree scale: 0.1

Supplementary Figure 15. Phylogeny of FtsZ homologues in Bacteria and Archaea. Maximum likelihood tree of FtsZ inferred with IQ-TREE v1.6.7.2 (ModelFinder best-fit model LG+R10) [8, 9] from an alignment of 429 sequences and 422 amino acid positions. Numbers at nodes represent ultrafast bootstrap supports [10]. The scale bar represents the average number of substitutions per site. The internal topologies roughly recapitulate known phylogenetic relationships, suggesting that FtsZ was already present in the LUCA. The separation of the two archaeal FtsZ1 and FtsZ2 copies suggests that they arose from an early gene duplication.

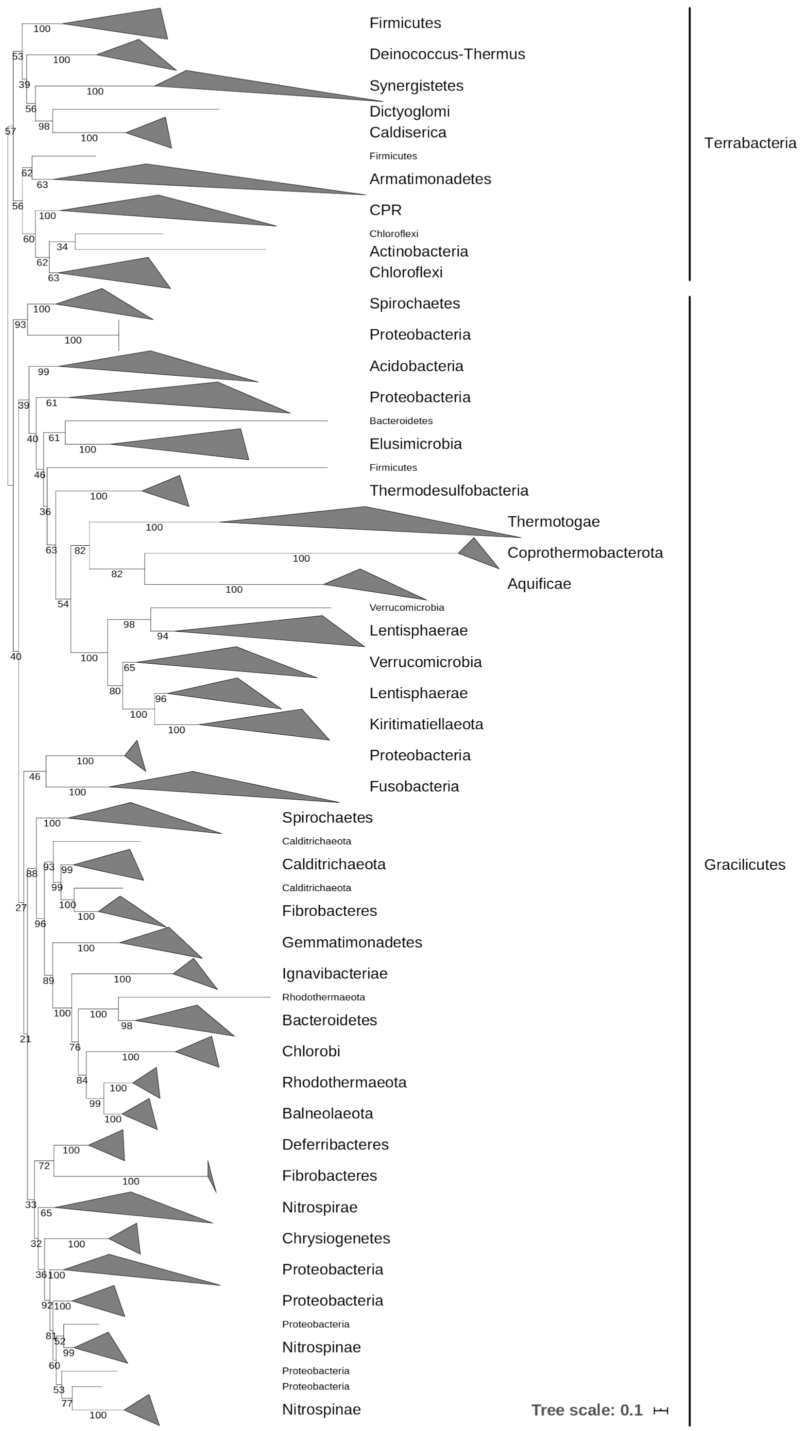

Supplementary Figure 16. Phylogeny of FtsA homologues in Bacteria.

Maximum likelihood tree of FtsA inferred with IQ-TREE v1.6.7.2 (ModelFinder best-fit model LG+R6) [8, 9] from an alignment of 163 sequences and 426 amino acid positions. Numbers at nodes represent ultrafast bootstrap supports [10]. The scale bar represents the average number of substitutions per site. The tree roughly recapitulates known phylogenetic relationships, suggesting that FtsA was already present in the LBCA (Last Bacterial Common Ancestor).

Supplementary Table 1. Bacterial strains and plasmids used in this study.

Strain or plasmid	Characteristics	References
E. coli		
**DH5*α***	F- endA1 Φ80dlacZΔM15 Δ(lacZYA-argF)U169 recA1 relA1 hsdR17(rK–mK+) deoR supE44 thi-1 gyrA96 phoA λ– ; strain used for general cloning procedures	[11]
Top10	F- *mcrA* Δ(*mrr-hsd*RMS-*mcr*BC) Φ80*lac*ZΔM15 Δ *lac*X74 *rec*A1 *ara*D139 Δ(*araleu*)7697 *gal*U *gal*K *rps*L (StrR) *end*A1 *nup*G; strain used for general cloning procedures	
BL21(DE3)	F- ompT hsdSB(rB–mB–) gal dcm (DE3); host for protein production	[12]
M. wolfeii		
DSM 2970	Methanogenic, anaerobic, 60°C, type strain,	[13] [14]
M. smithii		
DSM 861	Methanogenic, anaerobic, 37°C, type strain	[15]
**Plasmids**		
**pET-15b**	T7 promotor, His-tag, multiple cloning sites (*Nde*I - *Bam*H I), lacI, AmpR	
**pET-15b_*peiW***	AmpR, pET derived for *M. wolfeii* peiW recombinant expression containing a N-terminal His-tag followed by a thrombin cleavage site	[16]
**pET-SUMO-*sepF*_full**	KanaR; pET derivate for *M.smithii* SepF recombinant expression containing a N-terminal His-tag followed by a SUMO protease cleavage site	This work
**pET-SUMO-*sepF*_core**	KanaR; pET derivate for *M.smithii* SepF (53-149) recombinant expression containing a N-terminal His-tag followed by a SUMO protease cleavage site	This work
**pET-SUMO-*ftsZ*_full**	KanaR; pET derivate for *M.smithii* FtsZ recombinant expression containing a N-terminal His-tag followed by a SUMO protease cleavage site	This work

Supplementary Table 2. Oligonucleotides used in this study

**Oligonucleotide**	**Sequence (5'→ 3') and properties ***
**Construction of pET-15b_*peiW***	
PeiWF2	AGGTGATCATATGGAAGTGGGGCTAAATG
PeiWR2	AACAACTCGAGCATGTCTCTGCCACAAAC
**Construction of pET-SUMO-*sepF*_full**	
Ms_SepF_full_f	**CGAACAGATTGGTGGC**ATGGGTTTCACTGATG
Ms_SepF_full_r	**GTTAGCAGCCGGATCT**CTACTTTCTAACTAAACTGACTCC
**Construction of pET-SUMO-*sepF*_core**	
Ms_SepF_core_f	**CGAACAGATTGGTGGC**GATGATGTGTCTATTTCTCC
Ms_SepF_full_r	**GTTAGCAGCCGGATCT**CTACTTTCTAACTAAACTGACTCC
**Construction of pET-SUMO-*ftsZ*_full**	
Ms_FtsZ_full_f	**CGAACAGATTGGTGGC**GTGAAATTTATAGATGATGC
Ms_FtsZ_full_r	**GTTAGCAGCCGGATCT**TTAGAATATTCCATCAATGAAATC
**Primers for colony PCR used during the construction of the pET-15b_*peiW* plasmid**	
T7	TAATACGACTCACTATAGGG
T7_term	CTAGTTATTGCTCAGCGGT
**Primers for colony PCR used during the construction of the different pET-SUMO plasmids**	
188_pAW-27	CCCGCGAAATTAATACGACTCAC
187_pAW-26	CCTCAAGACCCGTTTAGAGGCC
*Overlaps for Gibson assembly are written in bold letters. Restriction sites are underlined.	

Supplementary Table 3. SIMcheck results

**Concerning cell from figure 2a right panel**			
**Check**	**Statistic**	**Value**	**Pass**
CIP*	C1 total intensity variation (%)	23.7	Yes
CIP*	C2 total intensity variation (%)	6.07	Yes
MCN[+]	C1 average feature MCNR	19.3	Yes
MCN[+]	C2 average feature MCNR	19.7	Yes
RIH[#]	C1 max-to-min intensity ratio	6,7	Yes
RIH[#]	C2 max-to-min intensity ratio	14.1	Yes
**Concerning cell from figure 2a left panel**			
**Check**	**Statistic**	**Value**	**Pass**
CIP*	C1 total intensity variation (%)	8.84	Yes
CIP*	C2 total intensity variation (%)	14.7	Yes
MCN[+]	C1 average feature MCNR	27.6	Yes
MCN[+]	C2 average feature MCNR	37	Yes
RIH[#]	C1 max-to-min intensity ratio	8	Yes
RIH[#]	C2 max-to-min intensity ratio	9.6	Yes

* Channel Intensity Profiles; total intensity variation > ~50% over the 9-z-window used to reconstruct each z-section may cause artifacts
[+] Modulation contrast-to-noise ratio (MCNR) image; inadequate (<4), low to moderate (4-8), good (8-12), very good-excellent (>12)
[#] Reconstructed Intensity Histogram; max-to-min intensity ratio, MMR <3 is inadequate, 3-6 is low, 6-12 is good, >12 excellent

Supplementary references

1. Gautier, R., Douguet, D., Antonny, B. & Drin, G. HELIQUEST: a web server to screen sequences with specific -helical properties. *Bioinformatics* **24**, 2101–2102 (2008).
2. Mészáros, B., Erdos, G. & Dosztányi, Z. IUPred2A: context-dependent prediction of protein disorder as a function of redox state and protein binding. *Nucleic Acids Res.* **46**, W329–W337 (2018).
3. Jones, D. T. Protein secondary structure prediction based on position-specific scoring matrices. *J. Mol. Biol.* **292**, 195–202 (1999).
4. Zimmermann, L. *et al.* A Completely Reimplemented MPI Bioinformatics Toolkit with a New HHpred Server at its Core. *J. Mol. Biol.* **430**, 2237–2243 (2018).
5. Duman, R. *et al.* Structural and genetic analyses reveal the protein SepF as a new membrane anchor for the Z ring. *Proc. Natl. Acad. Sci.* **110**, E4601–E4610 (2013).
6. Robert, X. & Gouet, P. Deciphering key features in protein structures with the new ENDscript server. *Nucleic Acids Res.* **42**, (2014).
7. Goldenberg, O., Erez, E., Nimrod, G. & Ben-Tal, N. The ConSurf-DB: pre-calculated evolutionary conservation profiles of protein structures. *Nucleic Acids Res.* **37**, D323-7 (2009).
8. Nguyen, L.-T., Schmidt, H. A., von Haeseler, A. & Minh, B. Q. IQ-TREE: a fast and effective stochastic algorithm for estimating maximum-likelihood phylogenies. *Mol. Biol. Evol.* **32**, 268–74 (2015).
9. Kalyaanamoorthy, S., Minh, B. Q., Wong, T. K. F., Von Haeseler, A. & Jermiin, L. S. ModelFinder: Fast model selection for accurate phylogenetic estimates. *Nat. Methods* **14**, 587–589 (2017).

10. Minh, B. Q., Nguyen, M. A. T. & Von Haeseler, A. Ultrafast approximation for phylogenetic bootstrap. *Mol. Biol. Evol.* **30**, 1188–1195 (2013).

11. Hanahan, D. Studies on transformation of Escherichia coli with plasmids. *J. Mol. Biol.* **166**, 557–580 (1983).

12. Studier, F. W. & Moffatt, B. A. Use of bacteriophage T7 RNA polymerase to direct selective high-level expression of cloned genes. *J. Mol. Biol.* **189**, 113–130 (1986).

13. Winter, J. *et al.* Methanobacterium wolfei, sp. nov., a New Tungsten-Requiring, Thermophilic, Autotrophic Methanogen. *Syst. Appl. Microbiol.* **5**, 457–466 (1984).

14. Wasserfallen, A., Nölling, J., Pfister, P., Reeve, J. & De Macario, E. C. Phylogenetic analysis of 18 thermophilic Methanobacterium isolates supports the proposals to create a new genus, Methanothermobacter gen. nov., and to reclassify several isolates in three species, Methanothermobacter thermautotrophicus comb. nov., Methanothermobacter wolfeii comb. nov., and Methanothermobacter marburgensis sp. nov. *Int. J. Syst. Evol. Microbiol.* **50**, 43–53 (2000).

15. Balch, W. E., Fox, G. E., Magrum, L. J., Woese, C. R. & Wolfe, R. S. Methanogens: reevaluation of a unique biological group. *Microbiol. Mol. Biol. Rev.* **43**, (1979).

16. Nakamura, K. *et al.* Application of pseudomurein endoisopeptidase to fluorescence in situ hybridization of methanogens within the family Methanobacteriaceae. *Appl. Environ. Microbiol.* **72**, 6907–6913 (2006).
