# Supplementary material for: SepF is the FtsZ anchor in archaea, with features of an ancestral cell division system
Source: Nat Commun. 2021 Jun 4;12:3214. doi: 10.1038/s41467-021-23099-8 (PMC8178401; doi:10.1038/s41467-021-23099-8)
Supplement: Supplementary file 3 — Description of Additional Supplementary Files [file 41467_2021_23099_MOESM3_ESM.pdf]

Description of Additional Supplementary Files

File Name: Supplementary Data 1
Description: TaxIDs with their phylogenetic classification used for the plotting of the presence and absence of FtsZ, SepF, FtsA and ESCRT-III on the reference phylogeny of archaea, including the protein accession numbers when the protein was present.

File Name: Supplementary Data 2
Description: TaxIDs with their phylogenetic classification used for the plotting of the presence and absence of FtsZ, SepF, FtsA and ESCRT-III on the reference phylogeny of bacteria, including the protein accession numbers when the protein was present.

File Name: Supplementary Movie 1
Description: Movie of a non-constricting *M. smithii* cell stained with anti-MsSepF (cyan) and anti-MsFtsZ antibodies (magenta) imaged by 3D SIM. Movie corresponds to images from Figure 1a left panel. MsFtsZ forms a patchy ring-like structure at mid-cell that largely overlaps MsSepF.

File Name: Supplementary Movie 2
Description: Movie of a constricting *M. smithii* cell stained with anti-MsSepF (cyan) and anti-MsFtsZ antibodies (magenta) imaged by 3D SIM. Movie corresponds to images from Figure 1a right panel. A smaller MsFtsZ ring corresponds to the almost completed cell constriction and two new rings are in the future division plane of the perspective daughter cells, with MsSepF forming discontinuous arcs that largely overlap with MsFtsZ.
