# Supplementary material for: SepF is the FtsZ anchor in archaea, with features of an ancestral cell division system
Source: Nat Commun. 2021 Jun 4;12:3214. doi: 10.1038/s41467-021-23099-8 (PMC8178401; doi:10.1038/s41467-021-23099-8)
Supplement: Supplementary file 8 — Reporting Summary [file 41467_2021_23099_MOESM8_ESM.pdf]

nature research

Corresponding author(s): Simonetta Gribaldo and Anne-Marie Wehenkel

Last updated by author(s): 2021/03/30

Reporting Summary

Nature Research wishes to improve the reproducibility of the work that we publish. This form provides structure for consistency and transparency in reporting. For further information on Nature Research policies, see our Editorial Policies and the Editorial Policy Checklist.

Statistics

For all statistical analyses, confirm that the following items are present in the figure legend, table legend, main text, or Methods section.

n/a	Confirmed	
☐	☒	The exact sample size (*n*) for each experimental group/condition, given as a discrete number and unit of measurement
☐	☒	A statement on whether measurements were taken from distinct samples or whether the same sample was measured repeatedly
☐	☒	The statistical test(s) used AND whether they are one- or two-sided<br>*Only common tests should be described solely by name; describe more complex techniques in the Methods section.*
☒	☐	A description of all covariates tested
☒	☐	A description of any assumptions or corrections, such as tests of normality and adjustment for multiple comparisons
☐	☒	A full description of the statistical parameters including central tendency (e.g. means) or other basic estimates (e.g. regression coefficient) AND variation (e.g. standard deviation) or associated estimates of uncertainty (e.g. confidence intervals)
☐	☒	For null hypothesis testing, the test statistic (e.g. *F*, *t*, *r*) with confidence intervals, effect sizes, degrees of freedom and *P* value noted<br>*Give P values as exact values whenever suitable.*
☒	☐	For Bayesian analysis, information on the choice of priors and Markov chain Monte Carlo settings
☒	☐	For hierarchical and complex designs, identification of the appropriate level for tests and full reporting of outcomes
☒	☐	Estimates of effect sizes (e.g. Cohen's *d*, Pearson's *r*), indicating how they were calculated

Our web collection on statistics for biologists contains articles on many of the points above.

Software and code

Policy information about availability of computer code

Data collection	For quantitative analysis and 3D SIM of immuno-labelled cells images were acquired by the ZEN software (Carl Zeiss, Germany).<br>Negative stain EM images were acquired by SerialEM: Digitized on Tecnai 12.<br>X-ray diffraction data were collected at 100K using beamlines Proxima-1 and Proxima-2 at the Soleil synchrotron (GIF-sur-YVETTE, France) and with the software MxCube.<br>Chemidoc (Biorad) for Western Blot and gel imaging
Data analysis	For X-ray crystallography data analysis we used: Phaser 2.7.17, Coot 0.9.4, Buster 2.10.4, XDS, AIMLESS, CCP4 suite and Chimera<br>Sequences were aligned using MAFFT 7.419 with the L-INS-i algorithm.<br>Alignments were trimmed with trimAl 1.2rev59.<br>Conservation of FtsZ C-terminal domain was represented with WebLogo (https://weblogo.berkeley.edu/logo.cgi).<br>Secondary structures were detected using Ali2D (online tool: toolkit.tuebingen.mpg.de/tools/ali2d).<br>For structure-based SepF alignment T-Coffee (http://tcoffee.crg.cat/) was used and graphical representation was made using ENDscript server (https://endscript.ibcp.fr/ESPript/ENDscript/).<br>HMM profiles and HMM-based homology searches were done with the HMMER 3.2.1 package.<br>Phylogenetic trees were reconstructed with IQ-TREE 1.6.7.2.<br>Gene absences were checked with TBLASTN (BLAST 2.6.0).<br>Phylogenetic tree figures were generated with iTOL (online tool: itol.embl.de).<br>Protein families were assembled using BLASTP (BLAST 2.6.0) and SILIX 1.2.951.<br>Image Lab (Biorad, https://www.bio-rad.com/fr-fr/product/image-lab-software?ID=KRE6P5E8Z)<br>Electron microscopy images of negatively stained FtsZ filaments and FtsZ bundles were analyzed using Fiji (ImageJ, https://fiji.sc/)) Version 2.0.0-rc-68/1.52i. and the data were extracted into Microsoft Excel 2016. Boxplots were created by using Microsoft Excel 2016 with the Real Statistics Resource Pack for Excel 2016 (http://www.real-statistics.com). The statistical analysis was performed using DataLab version 4.0

(Epina GmbH, Pressbaum, Austria).
The Fiji plugin SIMcheck was used to analyze the quality of the acquisition and the processing of SIM images. For further image analysis of SIM image z stacks we used Fiji. The epifluorescence microscopy images acquired for the quantitative analysis were processed using Fiji in combination with plugin MicrobeJ Version 5.13l (Ducret et al, 2016)

For manuscripts utilizing custom algorithms or software that are central to the research but not yet described in published literature, software must be made available to editors and reviewers. We strongly encourage code deposition in a community repository (e.g. GitHub). See the Nature Research guidelines for submitting code & software for further information.

Data

Policy information about availability of data

All manuscripts must include a data availability statement. This statement should provide the following information, where applicable:

- Accession codes, unique identifiers, or web links for publicly available datasets
- A list of figures that have associated raw data
- A description of any restrictions on data availability

The crystallographic data is available from the Protein Data Bank (www.rcsb.org), under the accession numbers (PDB code) reported in Table 1. The source data corresponding to figures 1c, 2c and 2d, and supplementary Figures 2, 4a, 5a and 5b are provided as a Source Data file. The data used for phylogenetic analysis and alignments is found here: https://data.mendeley.com/datasets/pz8893jzgk/draft?a=5a5da375-729f-44b3-83a2-ca2adf6675d6 . All other data are available from the corresponding authors upon reasonable request.
Structure of 7AL1: https://doi.org/10.2210/pdb7AL1/pdb
Structure of 7AL2: https://doi.org/10.2210/pdb7AL2/pdb

Field-specific reporting

Please select the one below that is the best fit for your research. If you are not sure, read the appropriate sections before making your selection.

☒ Life sciences ☐ Behavioural & social sciences ☐ Ecological, evolutionary & environmental sciences

For a reference copy of the document with all sections, see nature.com/documents/nr-reporting-summary-flat.pdf

Life sciences study design

All studies must disclose on these points even when the disclosure is negative.

Sample size	For the quantitative analysis of immunolabelled cells the sample size was >700 cells. The number of measured FtsZ filaments and bundles was 130 each.
Data exclusions	no data were excluded
Replication	All replicates were done in identical conditions and performed in duplicates or triplicates
Randomization	n.a.
Blinding	n.a.

Reporting for specific materials, systems and methods

We require information from authors about some types of materials, experimental systems and methods used in many studies. Here, indicate whether each material, system or method listed is relevant to your study. If you are not sure if a list item applies to your research, read the appropriate section before selecting a response.

Materials & experimental systems

n/a	Involved in the study	
☐	☒	Antibodies
☒	☐	Eukaryotic cell lines
☒	☐	Palaeontology and archaeology
☒	☐	Animals and other organisms
☒	☐	Human research participants
☒	☐	Clinical data
☒	☐	Dual use research of concern

Methods

n/a	Involved in the study	
☒	☐	ChIP-seq
☒	☐	Flow cytometry
☒	☐	MRI-based neuroimaging

Antibodies

Antibodies used	Purified MsSepFcore protein was used to raise polyclonal antibodies in guinea pig (Covalab).

Two peptides (CENAENGLEKLKSAADT and CGESDSGDRALESVHE) in equimolar amounts were used to raise polyclonal antibodies in rabbit against M. smithii FtsZ (Genosphere Biotech).
anti-rabbit horseradish peroxidase-linked antibody (Invitrogen, 31460)
anti-guinea pig horseradish peroxidase-linked antibody (Invitrogen, A18769)
anti-rabbit Alexa555-conjugated (Invitrogen, A27039)
anti-guinea pig Alexa488-conjugared (Invitrogen, A11073)

Validation

In order to validate the antibodies and test the expression of the proteins SepF and FtsZ in cells, crude extracts and purified recombinant SepF and FtsZ in serial dilution were loaded on a 4-12 % (w/v) Bis-Tris PAGE gel (Invitrogen) and Western blots were performed. For both, anti-MsSepF antibody and anti-MsFtsZ antibody the dilution used was 1:500. For the secondary antibodies, the anti-rabbit horseradish peroxidase-linked antibody for FtsZ and the anti-guinea pig horseradish peroxidase-linked antibody for SepF (Invitrogen), For both the dilution was 1:5000. Both antibodies, either raised against MsSepF core or the FtsZ peptide antibody specifically recognized bands of the correct size in whole cell extracts and on purified proteins.
